# Screening of the Anti-Neurodegenerative Activity of Caffeic Acid after Introduction into Inorganic Metal Delivery Systems to Increase Its Solubility as the Result of a Mechanosynthetic Approach

**DOI:** 10.3390/ijms24119218

**Published:** 2023-05-24

**Authors:** Anna Stasiłowicz-Krzemień, Natalia Rosiak, Andrzej Miklaszewski, Judyta Cielecka-Piontek

**Affiliations:** 1 Department of Pharmacognosy and Biomaterials, Faculty of Pharmacy, Poznan University of Medical Sciences, Rokietnicka 3, 60-806 Poznan, Poland; astasilowicz@ump.edu.pl (A.S.-K.); nrosiak@ump.edu.pl (N.R.); 2 Institute of Materials Science and Engineering, Poznan University of Technology, Jana Pawla II 24, 61-138 Poznan, Poland; andrzej.miklaszewski@put.poznan.pl

**Keywords:** caffeic acid, delivery system, acetylcholinesterase, butyrylcholinesterase, neuroprotection

## Abstract

The proven anti-neurodegenerative properties of caffeic acid in vivo are limited due to its poor solubility, which limits bioavailability. Therefore, caffeic acid delivery systems have been developed to improve caffeic acid solubility. Solid dispersions of caffeic acid and magnesium aluminometasilicate (Neusilin US2—Neu) were prepared using the ball milling and freeze-drying techniques. The solid dispersions of caffeic acid:Neu obtained by ball milling in a 1:1 mass ratio turned out to be the most effective. The identity of the studied system in comparison to the physical mixture was confirmed using the X-Ray Powder Diffractionand Fourier-transform infrared spectroscopy techniques. For caffeic acid with improved solubility, screening tests were carried out to assess its anti-neurodegenerative effect. The obtained results on the inhibition of acetylcholinesterase, butyrylcholinesterase, tyrosinase, and antioxidant potential provide evidence for improvement of caffeic acid’s anti-neurodegenerative activity. As a result of in silico studies, we estimated which caffeic acid domains were involved in interactions with enzymes showing expression relevant to the neuroprotective activity. Importantly, the confirmed improvement in permeability of the soluble version of caffeic acid through membranes simulating the walls of the gastrointestinal tract and blood-brain barrier further strengthen the credibility of the results of in vivo anti-neurodegenerative screening tests.

## 1. Introduction

Caffeic acid (CA) is a hydroxycinnamic acid derivative found in many plant families, such as *Asteraceae*, *Lamiaceae*, *Solanaceae*, *Umbelliferae*, and *Caprifoliaceae*. CA is present in various fruits, vegetables, and spices such as rosemary, thyme, sage, and tarragon, as well as in tea and coffee [1,2]. It demonstrates a wide variety of biological activities, expressed in antioxidant [3], immunomodulatory [4], antimicrobial [5], neuroprotective [6], anti-anxiolytic [7], antiproliferative [8], antidiabetic [9], and anti-inflammatory [10] properties.

One intriguing aspect is the neuroprotective potential of CA. As a result of in vitro studies, it has been determined that CA can chelate iron (II) ions (Fe^2+^) [11], scavenge hydroxyl radicals (OH٭), and reduce ions in the FRAP and CUPRAC [12] models. Moreover, its neuroprotective potential has been confirmed by the inhibition of acetylcholinesterase (AChE) [3,13] and butyrylcholinesterase [11] (BChE). CA has been shown to improve memory deficits in the inhibitory avoidance task [13,14] and to counteract L-methionine-induced memory deficit in rats. CA prevents induced loss of reductive capacity [6], cell damage, and oxidative damage [15], as it influences the levels of glutathione, catalase, lipid peroxidation, and superoxide dismutase. However, the neuroprotective potential of CA requires further studies in various biological models.

CA’s therapeutic potential is limited due to its poor solubility and oral bioavailability. Therefore, this study aimed to improve the physicochemical properties of CA and evaluate a possible relationship with the improvement of its biological activity. Considering the potential for neuroprotective action of CA, attempts have previously been made to improve its solubility in order to increase its bioavailability. Dispersions with hydrophilic carriers, encapsulation, and electrospinning have most often been obtained. CA has already been combined with cyclodextrins: α-cyclodextrin [16], β-cyclodextrin [16,17], hydroxypropyl-β-cyclodextrin [18,19], corn starch, and chitosan [20,21] with the use of a ground method in combination with liquid phases or with the use of iodine binding reaction. In addition, CA has been encapsulated within ethosomes [22] and solid lipid nanoparticles [22]. In [23], solid lipid nanoparticles were coated by chitosan, alginate, and pectin. In [24], electrospinning was used to prepare nanofibers with carob flour, whey protein concentrate, and polyethylene oxide. Chuysinuan et al. immobilized CA onto the surfaces of individual electrospun poly(l-lactic acid) fibers [25].

To the best of the authors’ knowledge, no attempts have been made to improve the solubility of CA by combining it with inorganic carriers such as amorphous magnesium aluminometasilicate.

To our best knowledge, this paper represents the first time that research has been reported on improving the solubility and biological activity of CA as a result of combining it with magnesium aluminometasilicate.

## 2. Results and Discussion

The CA systems were prepared by ball milling and freeze-drying in the mass ratios 1:1 and 1:3 with magnesium aluminometasilicate (Neusilin US2, Neu) and compared with systems with other carriers: Kollidon VA 64 (Koll.VA64), Eudragit L100 (Eud.L100), hydroxypropyl-β-cyclodextrin (HP-β-CD), Gohsenol EG-05PW (Goh). Physical mixtures were prepared as well. A 24-h solubility study was performed to determine the solid dispersion with the greatest potential for further studies.

CA solubility was determined as 303.274 ± 6.218 (μg/mL) (Table S1, Supplementary Materials). There was no statistical difference between this value and the CA solubility after freeze-drying or ball-milling without excipients. In the freeze-dried group, the best CA solubility was noticed in the system with Neu in the 1:3 mass ratio (2262.34 ± 14.766 μg/mL, see Table S1). However, the greatest improvement in CA solubility among all systems was noticed for 1:1 ball-milled solid dispersion with Neu—5449.017 ± 19.420 μg/mL. Thus, this system and its physical mixture were chosen for identification, physicochemical, and biological activity studies.

In the literature, in a study by Mangrulkar et al. [26], a CA solubility was determined as 0.68 ± 0.12 mg/mL; preparation of CA-Phospholipon^®^ 90H complex caused an increase in solubility to 2.87 ± 0.19 mg/mL (4.2-fold enhancement), while the physical mixture enlarged the solubility to 0.76 ± 0.15 mg/mL. In Sguizzato et al. [27], CA solubility was successfully increased by 1.76 times by a solid lipid nanoparticle preparation with respect to water, where CA water solubility was determined as up to 0.5 mg/mL. Water CA solubility in Kfoury et al. [28] was increased from 2.33 × 10^−3^ M (0.420 mg/mL) to about 9.5 × 10^−3^ M by combining it with α-cyclodextrin.

Attempts to improve the solubility by using amorphous magnesium aluminometasilicate with different compounds of natural origin have been reported previously, though not for CA. For example, Jha et al. [29] used amorphous magnesium aluminometasilicate during hot-melt extrusion of naringenin in combination with Poloxamer 188. As the result of the application of amorphous magnesium aluminometasilicate, improvements in curcumin [30] solubility and dissolution rate were observed after freeze-drying it with inulin.

X-ray powder diffraction (XRPD) and Fourier transform infrared spectroscopy (FT-IR) were used to assess the identity of the ball-milled system with Neu involved in such an excellent improvement of CA solubility.

**Figure 1:**
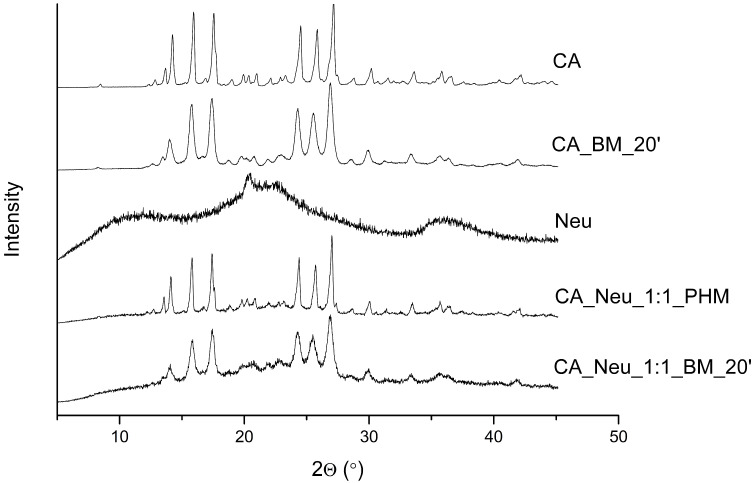
The X-Ray Powder Diffractionpatterns of caffeic acid (CA), 20 min ball-milled caffeic acid (CA_BM_20′), Neusilin US2 (Neu), CA and Neu physical mixture (CA_Neu_1:1_PHM), and solid dispersion obtained by 20 min of ball milling (CA_Neu_1:1_BM_20′).

XRPD CA pattern (Figure 1) shows several characteristic diffraction reflexes (13.6°, 14.2°, 15.9°, 17.5°, 24.5°, 25.8°, 27.1°, 30.1°, 33.5°, 35.8°) [31], proving crystal state of CA. The process of ball milling provided only for CA caused a slight decrease in reflex intensity; however, the crystalline state remained. Neu, on the other hand, is present in the amorphous state. The physical mixture of CA and Neu caused a slight decrease in the intensity of CA peaks. However, it was the process of ball milling for 20 min that caused the strongest intensity reduction in CA characteristic diffraction reflexes, which were widened, but still visible; thus, CA remained present in crystalline state, which could be attributed to the interactions between the two components. While XRPD did not yield amorphousness of CA, it did indicate a tendency towards it, which cannot be discounted as having an impact on the physicochemical properties of CA.

A Fourier-Transform Infrared Spectroscopy (FTIR) study supported by a Density Functional Theory (DFT) approach was performed to determine the interactions between Neu and CA at a structural level. DFT was used as a tool to allow even very subtle changes to be noticed in the infrared spectra, as these may have caused the improvement in the solubility of CA. The optimized geometry of the CA and the experimental and calculated spectra of CA are shown in Figure S1 (Supplementary Materials).

**Figure 2:**
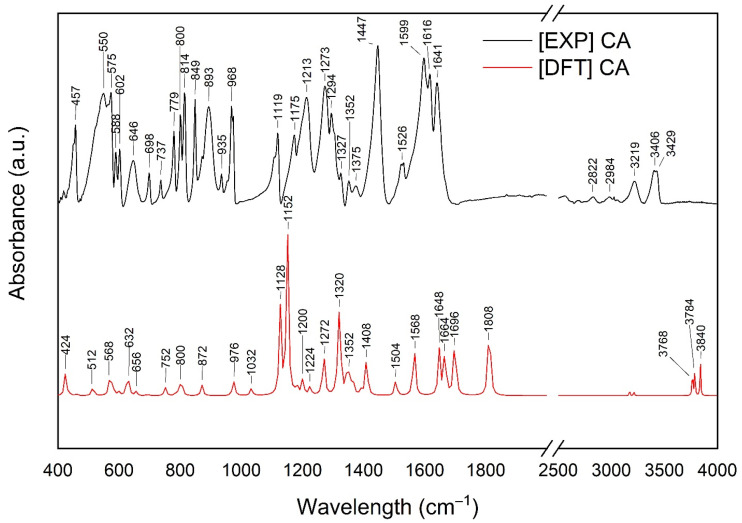
The experimental (EXP) Fourier-transform infrared spectroscopy (FT-IR) results of caffeic acid (CA) were compared with calculations (Density Functional Theory–DFT) obtained using a basis set 6–311G(d,p); range 400–4000 cm^−1^.

The wavenumber shifts between experimental and calculations spectra are expected and result from approximations used in the computational procedure. Most characteristic peaks of CA are described in Table S2 (Supplementary Materials). Bands corresponding only to the phenolic group (Figure S1) in CA were observed at about 457 cm^−1^, 849 cm^−1^, 1173 cm^−1^, 1375 cm^−1^, 1477 cm^−1^, 3402 cm^−1^, and 3428 cm^−1^ (Figure 2).

**Figure 3:**
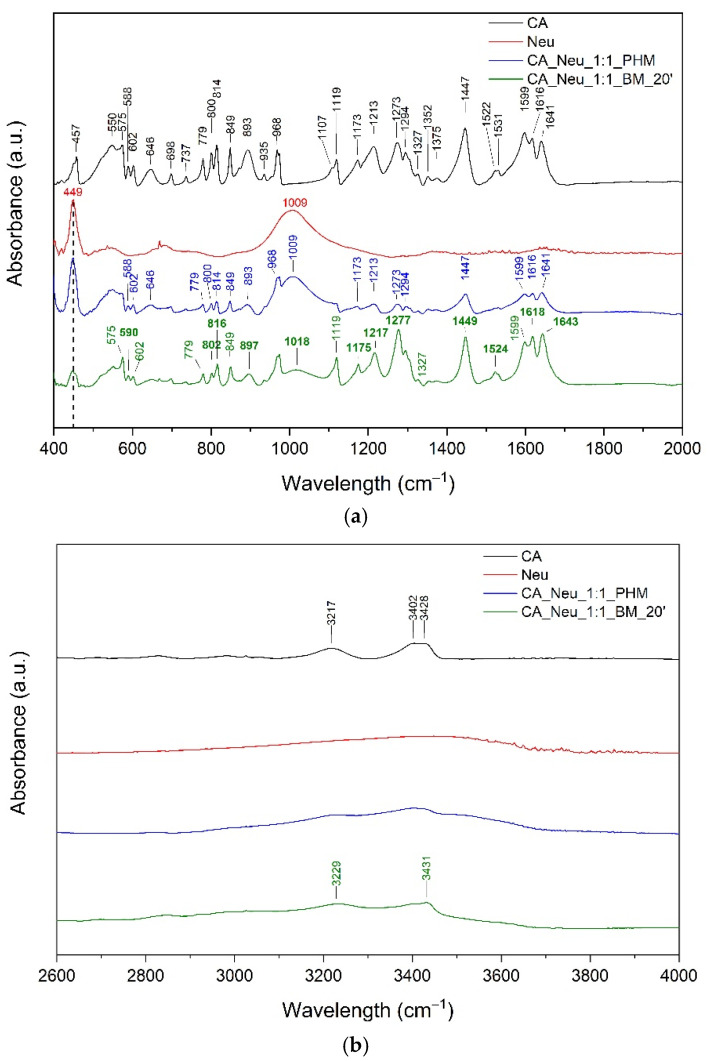
The Fourier-transform infrared spectra: Caffeic acid (CA—black line), Neusilin US2 (Neu—red line), CA and Neu physical mixture (CA_Neu_1:1_PHM—blue line), and solid dispersion obtained by 20 min of ball milling (CA_Neu_1:1_BM_20′—green line); range 400–2000 cm^−1^ (**a**) and 2600–4000 cm^−1^ (**b**).

Next, bands corresponding to the O–H group were observed at about 457 cm^−1^, 3402 cm^−1^, and 3428 cm^−1^. The band at 849 cm^−1^ can be assigned to C–OH stretching, CCC stretching, and/or C–H wagging. In addition, the band at 1173 cm^−1^ is attributed to C–O–H stretching, C–H wagging, and CC stretching, whereas the band observed at 1375 cm^−1^ is attributed to CC stretching and O–H bending. Intense bands at about 1477 cm^−1^ correspond to CC stretching in the phenolic ring. The unsaturated carboxylic acid chain bands corresponding to the O–H group were located at about 602 cm^−1^, 935 cm^−1^, 1119 cm^−1^, 1213 cm^−1^, 1294 cm^−1^, 1352 cm^−1^, and 3217 cm^−1^. In addition, bands attributed to the C–O–H were observed at 800 cm^−1^, 814 cm^−1^, and 1273 cm^−1^. The bands corresponding to the C–H group were present at 602 cm^−1^, 893 cm^−1^, 968 cm^−1^, 1213 cm^−1^, and 1294 cm^−1^. C=C stretching predominated at the range 1522–1616 cm^−1^. Neu showed characteristic signals at 449 cm^−1^ (O–Si–O bending), 1009 cm^−1^ (Si–O–Si stretching), and 3473.91 cm^−1^ (O–H stretching of the silanol group) [32,33,34]. The infrared spectrum of the ball-milled solid dispersion was analyzed and compared with the spectra of the pure component and the physical mixture (ph.m.) (see Figure 3a,b).

The spectrum recorded for CA ball-milled solid dispersion indicates changes in many characteristic bands. In the case of CA, the changes mainly involve bands associated with the carboxyl group. Among other things, we can point out band shifts at about 588 cm^−1^, 800 cm^−1^, 814 cm^−1^, 1213 cm^−1^, and 1273 cm^−1^ (see Figure 3a) assigned to the –COOH, C–O–H, or O–H group in the side chain (see Table S2, Supplementary Materials). These bands have additional components, for example, C–H vibrations. Furthermore, the bands at about 893 cm^−1^ and 1173 cm^−1^ attributed to the C–H group of CA in the CA–Neu systems are shifted to higher wavelengths. The same changes affect the characteristic bands of the C=C and C=O groups, with a shift in the CA bands at 1616 cm^−1^ (C=C stretching) and 1641 cm^−1^ (C=O stretching) and a decrease in peak intensity at 1599 cm^−1^ (C=C stretching) being indicated.

The observed changes suggest the formation of salts. It is indicated that changes observed in CA at 1616 cm^−1^ (C=C stretching), 1641 cm^−1^ (C=O stretching), and 1599 cm^−1^ (C=C stretching) confirm the formation of Si–O–C bridging bond between CA and Neu. It is not excluded that salt is formed by the interaction between the COO– moiety of CA and Mg^2+^ or Al^3+^. The literature confirms salt formation with Ney and silica materials [35,36,37]. Krupa et al. [38] reported the formation of a salt of ibuprofen with Neu. Additionally, Uegaki et al. [36] confirmed salt formation between indomethacin (IND) and porous calcium silicate (PCS). They confirmed interactions between the COO– moiety of IND and Ca^2+^ of PCS. Doan V et al. [37] described that the carbonyl band of –COOH shifts upon conversion to the Na salt form. Based on the alterations in FTIR spectra, Mallick et al. [35] suggested an acid–base reaction between the Al_2_O_3_ (hydrated aluminium silicate) in kaolin and the carboxylic acid in ibuprofen to form the salt. In another paper, Kararli et al. [39] reported an acid–base reaction between MgO and ibuprofen, which resulted in the formation of a magnesium salt of ibuprofen in the solid state. In addition, they suggested that water mediates the acid–base reaction between the crystalline forms of MgO and ibuprofen. Therefore, in the case of Neu, it is possible to form salts with CA both through interactions with Mg^2+^ or Al^3+^. It is not excluded that water molecules (present in Neu) can mediate the acid-base reaction resulting in the formation of salt.

In addition, changes in CA bonds were observed in the spectrum of ball-milled solid dispersion with Neu in the range 800–900 cm^−1^ (C–H, C–O–H, C–C–C), 1175–1280 cm^−1^ (C–O–H, C–H, C–C), and 3000–3450 cm^−1^ (–OH stretching) (see Figure 3a,b and Supplementary Table S2). These results confirm the intermolecular hydrogen bonds between the O–H or C–H group in CA and the O–Si group in Neu. Gupta et al. [40] have previously documented the occurrence of hydrogen bonds when co-grinding Neu with the non-steroidal anti-inflammatory drugs indomethacin, ketoprofen, and naproxen, all of which contain carboxylic acid. Shifting wavelengths in the range above 3000 cm^−1^ were reported by Vadher et al. [41] for aceclofenac–Neu grinding mixtures.

The FT-IR spectrum of the physical mixture is the superposition of bands originating from CA and Neu. In addition, no band shifts characteristic of CA were observed, which confirmed no interaction between CA and Neu in the physical mixture.

**Figure 4:**
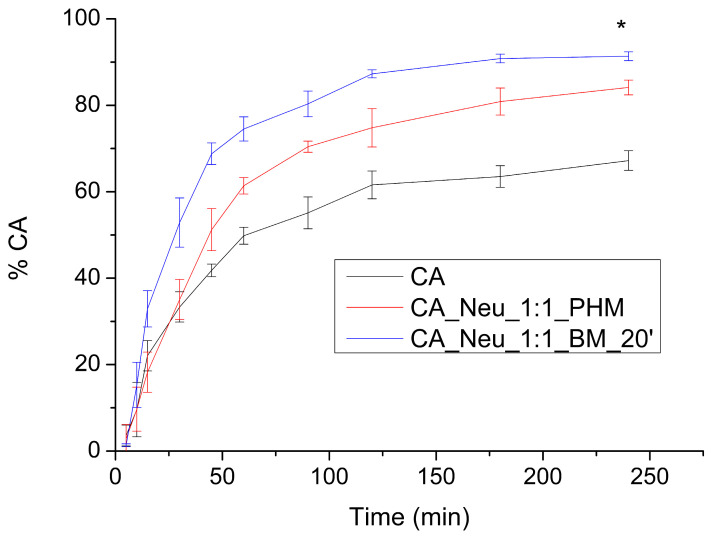
Apparent solubility of caffeic acid (CA), CA and Neusilin US2 (Neu) physical mixture (CA_Neu_1:1_PHM), and solid dispersion obtained by 20 min of ball milling (CA_Neu_1:1_BM_20′) at a pH of 1.2. (*) indicates statistically significant differences.

The apparent solubility study was carried out in two pH conditions resembling the conditions present in the gastrointestinal tract (GIT), namely, pH values of 1.2 and 6.8. At pH 1.2 (Figure 4) over 30 min, pure CA was dissolved in 33.435 ± 3.495%, while in a physical mixture CA was dissolved in 35.051 ± 4.637%. The greatest difference was observed for the CA_Neu_1:1_BM_20′ solid dispersion, in which CA reached a dissolution rate of 52.882 ± 5.698%. After 90 min, CA was dissolved in 55.106 ± 3.685%, the physical mixture in 70.425 ± 1.294%, and in the ball milled solid dispersion in 80.333 ± 2.972%. The highest dissolution rate of CA was obtained in 240 min at the level of 67.213 ± 2.492%, 84.136 ± 1.714%, and 91.360 ± 1.013% for pure CA, CA in the physical mixture, and in the solid dispersion obtained by ball milling, respectively. Not only did ball milling and the addition of Neu cause an increase in dissolution rate, it fastened the dissolution of CA significantly, as the dissolution profiles were assessed as different using fit factors.

**Figure 5:**
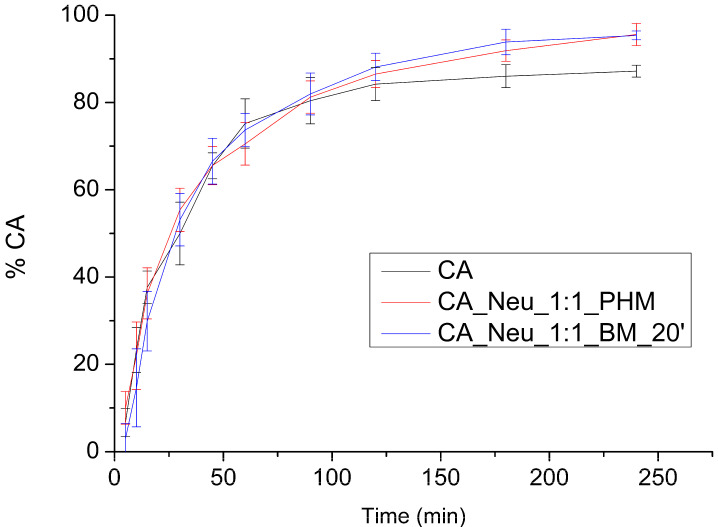
Apparent solubility of caffeic acid (CA), CA and Neusilin US2 (Neu) physical mixture (CA_Neu_1:1_PHM). and solid dispersion obtained by 20 min of ball milling (CA_Neu_1:1_BM_20′) in a pH of 6.8.

At pH 6.8 (Figure 5), the CA dissolution profile was similar to CA in the physical mixture and ball-milled system. The highest dissolution rate was reached for CA after 240 min (87.171 ± 1.353%); the highest dissolution noted in the physical mixture was 95.576 ± 2.534%, and for the milled system it was 84.251 ± 1.013%. The differences between dissolution profiles at this pH are less visible than for pH 1.2. The greatest variance is noted between CA and combinations with Neu at 180 and 240 min.

Fathi et al. performed a study in which CA-loaded solid lipid nanoparticles were electrostatically coated with chitosan, alginate, and pectin in different concentrations [23]. Pectin–chitosan-coated solid lipid nanoparticles demonstrated superior performance in retarding the release of CA in gastric media, up to 2.5 times higher than uncoated solid lipid nanoparticles. Vertuccio et al. [42] prepared hybrid systems consisting of inorganic/organic silica, polyethylene glycol, and CA using the sol-gel method, with varied weight percentages of polyethylene glycol and CA in the synthesis process. In a kinetic release study in simulated body fluid at 37 °C, CA was released in two steps: rapid dissolution, and diffusion from the material surface followed by slower dissolution within the material clusters. An increase in the amount of polyethylene glycol led to a decrease in the release rate.

CA permeability through GIT walls was determined at pH 1.2 and 6.8 by the parallel artificial membrane permeability assay (PAMPA) model (Table 1).

In acidic conditions, the CA permeability coefficient was 7.405 × 10^−6^ ± 2.872 × 10^−7^ cm s^−1^, for the ball-milled system with Neu there was a statistically significant increase of CA permeability with a permeability coefficient of 8.850 × 10^−6^ ± 1.765 × 10^−7^ cm s^−1^, and for the physical mixture with Neu the results were 7.546 × 10^−6^ ± 7.741 × 10^−8^ cm s^−1^. In the gastric environment, CA is sufficiently permeable, as the permeability coefficient exceeds 1.000 × 10^−6^ cm s^−1^. However, at pH 6.8 the permeability is poor, assessed for CA as 1.445 × 10^−7^ ± 3.486 × 10^−8^ cm s^−1^, for the ball-milled system as 1.812 × 10^−7^ ± 8.183 × 10^−9^ cm s^−1^, and for the physical mixture as 1.322 ± 2.431 × 10^−8^ cm s^−1^ using the *P_app_* value.

Hydroxyl cinnamic acid derivatives [43] are absorbed well from the stomach and intestine, which is influenced by their structure compared to other complex phenolic compounds. CA can be passively absorbed in the stomach [44], as assessed within this study. However, most CA absorption takes place in the membranes of the intestinal cells [45]. In the Wang et al. study [46], 5 mg/kg of CA was administered into rats’ duodenums, where intestinal absorption was determined as 12.4%. In the Caco-2 cell model [46], the CA *P_app_* A→B values ranged from 4.87 ± 1.72 × 10^−7^ cm s^−1^ to 5.05 ± 0.66 × 10^−7^ cm s^−1^, while the studied CA concentration was 5 to 15 µg/mL. In studies from the literature on the impact on CA permeability, melatonin [47] did not alter Caco-2 CA permeability, while rats fed with coconut oil [48] presented higher absorption of CA.

The CA blood–brain barrier (BBB) passive permeability was studied only for CA, as Neu does not enter the bloodstream to reach the BBB. The apparent permeability coefficient was calculated as 1.142 × 10^−6^ ± 2.006 × 10^−7^ cm/s, which classifies CA as slightly permeable.

A study by Grabska-Kobylecka et al. [49] assessed the presence of twelve phenolics in the cerebrospinal fluid and in plasma samples collected from patients with neurological disorders who were undergoing diagnostic lumbar puncture. CA was detectable in the cerebrospinal fluid; however, its concentration did not correlate with its plasma concentrations, as CA present in human body fluids is not exclusively obtained from dietary sources. Moreover, the authors underline that CA transportation through the BBB is not the result of simple or facilitated diffusion.

The BBB is a highly selective and tightly regulated barrier that separates the circulating blood from the brain tissue. While the BBB serves a critical role in protecting the brain from harmful substances, it presents a challenge for the delivery of therapeutic agents, including certain compounds such as CA, into the brain. The endothelial cells that form the blood vessels in the brain are connected by tight junctions, which create a physical barrier that restricts the passage of molecules [50]. Another issue is that the BBB is equipped with efflux transporters, such as P-glycoprotein, that actively pump out various molecules from the brain back into the bloodstream [51]. Compounds that permeate BBB should not have a large structure, as peptides and proteins generally face greater difficulty in crossing the BBB due to their size. Small compounds such as glucose, amino acids, and nucleosides pass the BBB easily [52]. Overcoming these challenges and enhancing BBB permeability requires the development of specific strategies, such as prodrugs, nanoparticle-based delivery systems, penetration enhancers, or combination therapies to improve biocompatibility. This means overcoming all barriers on the way from oral administration to BBB, including absorption, circulation in the bloodstream, and optimization of BBB permeability [53,54,55]. Andrade et al. [56] loaded CA into liposomes and surface-modified them with transferrin to enhance delivery across the BBB. The optimized transferrin-modified liposomes demonstrated suitable size, stability, and sustained release of CA and exhibited anti-amyloidogenic effects by preventing Aβ aggregation and fibril formation. Because the BBB is most permeable to glucose, caffeic glucose derivatives may pass through the BBB via active transport. Proper compounds combined in the perfect ratio might impact BBB permeability while additionally providing neuroprotective activity. In Akomolafe et al. [57], caffeine, CA, and their combinations inhibited the activities of AChE, monoamine oxidase, ecto-nucleoside triphosphate diphosphohydrolase, and ecto-51-nucleotidase while stimulating Na^+^/K^+^ ATPase activity, suggesting that the proportional combination of these bioactive compounds is crucial to their anti-neurodegeneration potential.

Antioxidant activity is crucial for neuroprotection, as it helps to prevent the damage caused by reactive oxygen species (ROS) and other free radicals. Antioxidants work by neutralizing ROS and free radicals, preventing them from causing further damage to neurons and other cells in the nervous system. This leads to the reduction of inflammation associated with oxidative stress and neuronal damage. There are reports in the literature proving the neuroprotective effects of antioxidants in preclinical and clinical studies [58,59,60].

In the DPPH antioxidant activity study, the CA IC_50_ (concentration needed to decrease the initial radical concentration by 50%) was defined as 362.507 ± 2.623 μg/mL. At CA water solubility concentration (303.27 ± 6.22 μg/mL), DPPH is scavenged in 44.408 ± 0.568%. The reference substance, Trolox, showed a higher antioxidant activity—IC_50_ value DPPH—93.640 ± 1.072 µg/mL. After the introduction of CA to Neu in both physical mixture and ball-milled solid dispersion, the obtained water solubility exceeded the concentration needed for maximal scavenging of the radical (plateau), which is a statistically significant improvement. In the ABTS and FRAP models, CA IC_50_ and IC_0.5_ (concentration indicating 0.5 absorbance) were determined within CA water solubility; in ABTS, IC_50_ was 84.517 ± 0.735 μg/mL, and in FRAP it was 22.753 ± 0.702 μg/mL (for Trolox, the values were 120.188 ± 2.726 µg/mL and 41.941 ± 0.014 µg/mL, respectively), indicating that CA is a strong antioxidant.

In the DPPH assay performed by Shiozawa et al. [16] (with the use of DPPH 100 μM methanolic solution), the CA IC_50_ value was determined as 2.57 µg/mL. CA ground mixtures with α-CD and β-CD (molar ratio = 1/1) caused an increase in radical scavenging potential, decreasing IC_50_ values to 1.42 µg/mL for the ground mixture with α-CD, and 1.77 µg/mL for the ground mixture with β-CD. The difference in antioxidant activity between the ground mixtures was stated to be the result of differences in stability constants and structures of the inclusion complexes. The antioxidant properties of CA-loaded collagen and chitosan hydrogel composites [61] developed with the use of a solvent casting method were studied by DPPH, ABTS, and FRAP techniques. The most efficient activity was noted for 30% composite hydrogel, as 0.1 mM DPPH solution was inhibited by 84.59%, ABTS radical was scavenged at 49.38%, and Ferrum (Fe^3+^—TPTZ) was reduced by 0.93%. In Katuwavila et al. [62], CA-loaded liposomes were prepared using the reverse phase evaporation technique, and their permeability through dialysis membrane and pig ear skin were studied. Afterwards, permeated loaded CA was studied against DPPH radical. The dialysis membrane permeated loaded CA scavenged DPPH at 86.18%, while CA at the same concentration scavenged DPPH at 86.18%. After skin permeation, loaded CA antioxidant activity was assessed as 51.07%, while CA at the same concentration inhibited 56.66%. In addition, CA phenethyl ester was found to have potent biological activity. Tosheva et al. [63] designed micelles loaded with CA phenethyl ester based on a poly(ethylene oxide)-b-poly(ε-caprolactone)-b-poly(ethylene oxide) triblock copolymer and its derivatives with cinnamyl-modified segments. In a model of oxidative stress induced by H_2_O_2_, both pure CA phenethyl ester and loaded micelles provided significant protection against H_2_O_2_-induced damage, while micelles with cinnamyl-modified segments demonstrated superior antioxidant protection even at low concentrations compared to pure CA phenethyl ester and micelles without modifications.

Inhibition of AChE and BuChE can lead to an increase in the concentration of acetylcholine in the synaptic cleft, improving neurotransmission and cognitive function in Alzheimer’s disease and Parkinson’s disease [1]. The anti-neurodegenerative effects of AChE and BuChE inhibition are thought to be mediated by reducing amyloid-β deposition, enhancing neurotrophic factor expression, and modulating inflammatory responses [6]. AChE and BuChE inhibitors, such as rivastigmine and galantamine, improve cognitive function and reduce neuroinflammation in animal models and in patients [64,65,66]. The neuroprotective potential of CA is described in the literature. CA inhibits AChE partially purified from human serum during in vitro studies, with the IC_50_ determined as 16.80 ± 1.43 mM. In vitro CA impact on AChE studies [13] was followed by in vivo [13] studies where rats were administered 10 mg/kg or 50 mg/kg or 100 mg/kg of CA orally once a day for 30 days. After 30 days of treatment at all dosages, significantly inhibited AChE activity in the cerebral cortex and striatum and elevated enzyme activity in the hypothalamus, hippocampus, and pons was found. Intake of 100 mg/kg of CA improved memory in the inhibitory avoidance task in rats [13]. In another study [14], male Sprague-Dawley rats were treated with 20 and 40 mg/kg once a day for 28 days orally to study the impact of CA against L-methionine induced memory deficit. The effectiveness of CA was proven, as impairments of spatial and recognition memories were not present in CA-treated groups. In a study on PC12 cells [67] (rat pheochromocytoma cells), pretreatment with CA one hour prior to beta-amyloid peptide significantly reversed the induced neurotoxicity by counteracting the increase of intracellular calcium concentration and tau phosphorylation. Colonnello et al. [6] found that CA (100 μM) prevented induced loss of reductive capacity, cell damage, and oxidative damage in rat cortical slices. In wild-type [6] (N2) of *Caenorhabditis elegans*, CA (25 mM) protected against toxic insults and weakened the induced loss of survival and motor alterations. In rats, administration of CA (50 mg/kg p.o) daily four days before colchicine injection [15] (induction of sporadic model of Alzheimer’s disease) and summarily for 25 days resulted in significant counteraction of worsening of cognitive abilities and decrease of induced changes in levels of AChE, glutathione, catalase, lipid peroxidation, and superoxide dismutase. In vitro studies of CA showed the potential of BChE [11] inhibition, with the greatest results obtained in combination with donepezil 0.025 mg/mL + CA 0.075 mg/mL. Moreover, CA prevented induced lipid peroxidation in rat brain homogenate in the same combination, as did donepezil 0.050 mg/mL + CA 0.050 mg/mL. CA (30 mg/kg b.w./day for 30 weeks) reduced the effects of AD pathogenesis [68] and connected mechanisms in high-fat (HF) diet-induced hyperinsulinemic rats, decreased memory and learning impairments, improved the levels of superoxide dismutase and glutathione, and reduced the level of Aβ 1–42) in the hippocampus. In Saenno et al., rats treated with D-galactose experienced memory deficits and a decline in hippocampal neurogenesis [69]. However, administration of CA (20 or 40 mg/kg for eight weeks) attenuated these effects, suggesting that CA has the potential to alleviate memory impairment and promote neurogenesis in the hippocampus. Despite these results, the neuroprotective properties of CA continue to require further studies with different biological models.

Increasing the solubility of CA may lead to improvements in the bioavailability of CA [26] and its ability to reach effective concentrations for interacting with the active center of enzymes that play a crucial role in the development of neurodegeneration [70]. Therefore, we carried out AChE, BChE, and tyrosinase inhibition studies in CA water concentrations before and after system preparation.

AChE and BChE inhibition were assessed for CA at its water solubility (303.27 ± 6.22 μg/mL). No inhibition of either of these enzymes was noticed at this point (Table 2). CA at the concentration of CA solubility in ball-milled solid dispersion (5449.02 ± 19.420 μg/mL) inhibited the AChE enzyme at 18.444 ± 0.429%, which was statistically significant, while CA in physical mixture with Neu (3281.838 ± 15.283 μg/mL) inhibited AChE at 11.854 ± 0.396%, which was again statistically significant. The inhibition of BChe was elevated significantly by the combination of CA with Neu. The concentration obtained by the ball-milled solid dispersion inhibited BChE at 13.011 ± 0.209%, while the physical mixture reached 8.644 ± 0.121%.

In the study of tyrosinase inhibition, pure CA inhibited the enzyme at 2.657 ± 0.039%, while CA at the concentration of ball-milled solid dispersion with Neu inhibited the enzyme at 58.658 ± 0.173%, whereas the physical combination increased the inhibition of tyrosinase to 29.443 ± 0.150% (Table 2). The enlargements of tyrosinase inhibition were statistically significant.

**Figure 6:**
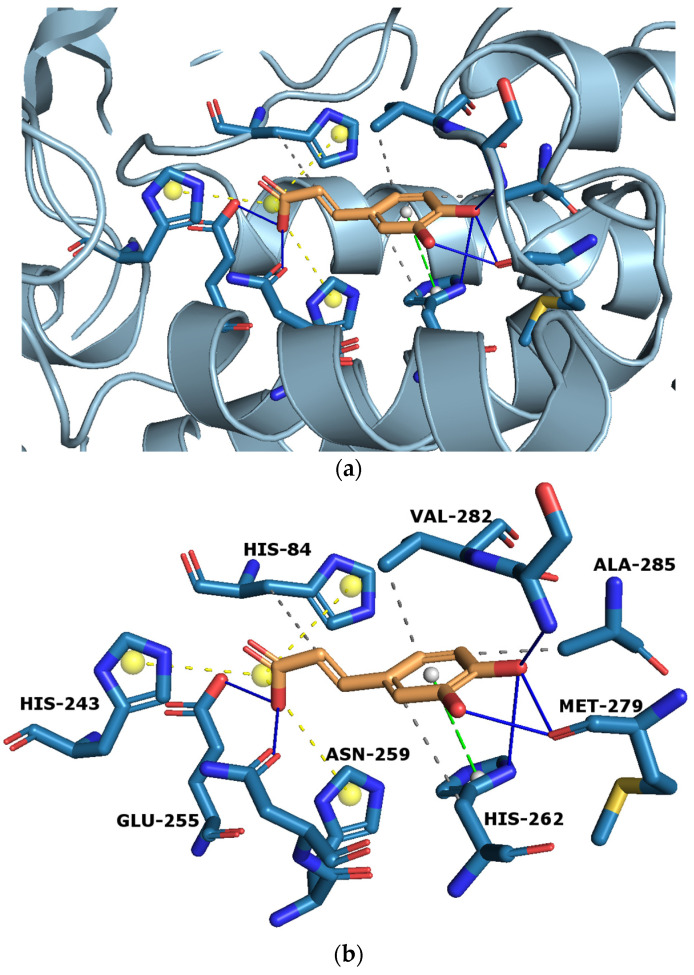
Computational docking simulation of binding between tyrosinase and caffeic acid (CA). (**a**) The corresponding surface structure of tyrosinase interacting with CA. (**b**) The interaction between CA and tyrosinase. The orange structure represents CA, while the blue structures show tyrosinase residues. Legend: solid blue line—hydrogen bonds, dashed green line—π-π interaction, dashed grey line—hydrophobic interactions, dashed yellow line—salt bridge.

The outstanding improvement of tyrosinase inhibition encouraged us to determine how CA interacts with the enzymes. The in silico method was used to establish the amino acid residues with which the active site of tyrosinase interacts. The selected docked conformation of CA in the tyrosinase (PDB ID: 2Y9X) binding site is shown in Figure 6a,b. The lowest binding energy was −8.07 kcal mol^−1^. As shown in Figure 6a, CA inserted in the active site of tyrosinase interacted with various amino acid residues, including Asn259, Glu255, His84, His262, His263, Met279, and Ser281. Moreover, in Figure 6b it can be observed that six hydrogen bonds were formed between the C-3, C-4, and C-9 hydroxyl groups of CA and five active-site residues Glu255, Asn259, His262, Met279, and Ser281, respectively. In addition, a π-π interaction between CA and His263 was found, along with four hydrophobic interactions with His84, His262, Val282, and Ala285.

Enzyme inhibitory activity by CA was described in the literature by both in vitro [71] and in vivo studies. In Işık and Beydemir [72], CA inhibition of partially purified AChE from human serum was determined and the molecular docking between CA and the enzyme was assessed. The IC_50_ value was determined as 16.80 ± 1.43 mM. With a docking score of 5.90 kcal/mol, CA showed a strong H-binding formation with Tyr124 at 4EY5 (1.97 Å). Moreover, CA exhibited hydrophobic interactions with Tyr119, Leu130, Tyr133, Phe297, Tyr337, and Phe338.

Anwar et al. [13] performed in vitro and in vivo AChE inhibition studies. Interestingly, CA at in vitro concentrations of 0.5, 1.0, 1.5, and 2 mM caused activation of the enzyme in the cerebral cortex, cerebellum, hypothalamus, whole blood, and lymphocytes, while in muscles CA inhibited AChE. However, in the in vivo part of the study in rats, CA at concentrations of 50 and 100 mg/kg administered by gavage for 30 days reduced AChE activity in the cerebral cortex and striatum while increasing activity in the cerebellum, hippocampus, hypothalamus, pons, lymphocytes, and muscles. According to the literature, CA and related compounds may have various effects on AChE in different tissues [72].

## 3. Materials and Methods

### 3.1. Materials

CA (purity > 98%) was supplied by TCI Chemicals (Tokyo, Japan). Neusilin US2 was kindly provided by Fuji Chemical Industry (Minato, Tokyo). Hydroxypropyl-β-cyclodextrin (molar substitution 0.8, Mw ~ 1.460) was purchased from Sigma-Aldrich (Poznan, Poland). Other excipients were kindly provided: Kollidon VA 64 by BASF Pharma (Florham Park, NJ, USA), Eudragit L100 by Evonik Industries (Essen, Germany), Gohsenol EG-05PW by Shin-Etsu Chemical (Tokyo, Japan). Acetonitrile (high-performance liquid chromatography [HPLC] grade) was provided by Merck (Darmstadt, Germany). Formic acid 98–100% was purchased from POCH (Gliwice, Poland). Hydrochloric acid, DMSO, NaCl, and potassium dihydrogen phosphate were supplied by Avantor Performance Materials (Gliwice, Poland). Acceptor sink buffer, Prisma HT, and GIT lipid solution were purchased from Pion Inc (Forest Row, East Sussex, England).

### 3.2. The Preparation of the CA Solid Dispersions

This study presents dispersion systems of CA obtained with amorphous magnesium aluminometasilicate, which were compared to similar CA systems with Kollidon VA 64, Eudragit L100, hydroxypropyl-β-cyclodextrin, and Gohsenol EG-05PW in a solubility study. All systems were prepared by ball milling and freeze-drying in the mass ratios of 1:1 and 1:3.

Solid dispersions of CA with excipient Neu and other excipients, such as hydroxypropyl-β-cyclodextrin, Kollidon VA 64, Eudragit L100, and Gohsenol EG-05PW, were prepared in mass ratios 1:1 and 1:3 by freeze-drying and ball milling (BM). BM was performed using a Retsch MM 400 mill (Retsch, Katowice, Poland) with steel jars filled with two steel balls (7.0 mm diameter) each. The frequency of rotations was set at 30 Hz. The jars were filled with CA, or with CA and excipients in a mass ratio of 1:1 and 1:3, and milled in 5 min time intervals (5 min of work and a five-minute break). After every 5 min milling, the sample was withdrawn and stored at room temperature. The total milling time was 20 min.

Freeze-drying was carried out with the use of the Heto PowerDry PL3000 Freeze Dryer (Thermo Scientific, Waltham, MA, USA). CA or mixtures of CA and excipients were weighed, dissolved, and suspended in MOH:H_2_O solution (the MOH:H_2_O ratio differed for each system, the as MOH concentration was intended to be as low as possible. The solutions were frozen in flasks at −20 °C (24 h) and then lyophilized. During the freeze-drying process, the temperature was maintained at −55 °C and the pressure was reduced to 6 hPa; the duration of the process was 72 h. The systems were stored at room temperature.

### 3.3. X-ray Powder Diffraction of the CA and CA Systems

The crystalline or amorphous nature of Ca, Neu, their 1:1 physical mixture, and the 20-min ball milled system was studied using the XRPD method. Diffraction patterns were obtained using a PANalitycal Empyrean diffractometer equipped with CuKα radiation (1.54056 Å).The tube voltage was set at 45 kV and the tube current at 40 mA. The angular range of measurement spanned from 3° to 50° with a step size of 0.017° and counting rate of 15 s/step. Data analysis was performed using OriginPro 8 software [73].

### 3.4. Fourier-Transform Infrared Spectroscopy of the CA and CA Systems

The spectra were measured using an IRTracer-100 spectrophotometer (Kyoto, Kyoto Prefecture, Japan) in absorbance mode, covering a frequency range from 4000 and 400 cm^−1^. The instrument was set with a resolution of 4 cm^−1^ with 400 scans and Happ-Genzel apodization. The samples were placed on the attenuated total reflectance (ATR) crystal and pressed against the ATR crystal while the ATR-FT-IR spectrum was scanned. Identification, intensity, and location of bands on IR spectrum of CA were determined by comparison with the theoretical FT-IR spectrum obtained through density functional theory (DFT) calculations. The geometry was optimized using DFT with Becke, three-parameter, Lee-Yang-Parr hybrid functional, and 6–311G(d,p) basis sets. DFT calculations were performed using the PL-Grid platform (website: www.plgrid.pl, accessed on 10 December 2022) and the Gaussian 09 package (Wallingford, CT, USA) [74,75]. The GaussView (Wallingford, CT, USA, Version E01) program was used to propose an initial geometry of the investigated molecules and for visual inspection of the normal modes [76]. The spectra of CA, Neu, their 1:1 mass physical mixture, and 20-min ball-milled solid dispersion were analyzed using Origin Pro 8 software (OriginLab Corporation, Northampton, MA, USA).

### 3.5. Chromatographic Conditions of the CA Separation

**Figure 7:**
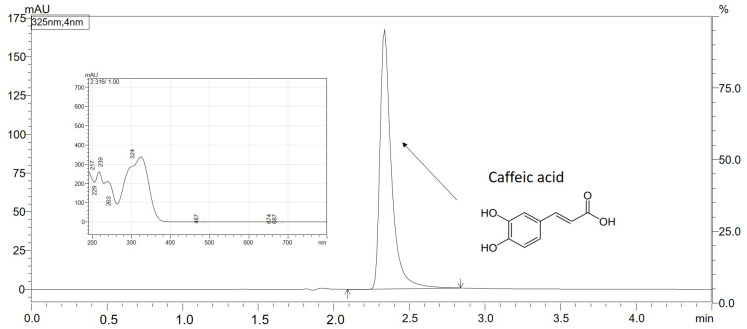
The chromatogram of caffeic acid (CA).

CA quantification of solubility, dissolution rate, and permeability studies was performed using high-performance liquid chromatography with the DAD detector (Shimadzu Nexera, Shimadzu Corp., Kyoto, Japan). The separation was achieved with the use of a Dr. Maisch ReproSil Chiral-JM-R C18 column (50 mm × 4.6 mm; 5 µm) (Dr. Maisch, Ammerbuch-Entringen, Germany) as a stationary phase and 0.1% formic acid/acetonitrile (70:30 *v/v*) as mobile phase. The CA retention time was about 2.33 min and the validated method duration time was 4.5 min. The temperature of the column was adjusted to 313.15 K, with a flow rate of 1.0 mL/min; an injection volume of 10 µL was used. Samples were scanned between 190–800 nm and integration was conducted at 325 nm. The results were obtained and processed by LabSolutions LC software (Shimadzu Corp., Kyoto, Japan) (Figure 7).

### 3.6. Solubility of CA in Pure Form and after Introduction into the Inorganic Metal Delivery System

To determine the solubility of the CA and CA systems, their excess amounts were placed in glass vials, and distilled water (5 mL) was pipetted. The vials were placed in a laboratory incubator MaxQ 4450 (Thermo Scientific, Waltham, MA, USA), kept for 24 h at 298.15 K, and shaken at a constant speed of 75 rotations per minute (rpm). After incubation, the obtained suspensions were filtered through 0.22 μm and studied by the high-performance liquid chromatography (HPLC) method. All measurements were performed in triplicate.

### 3.7. The Apparent Solubility of CA from the Inorganic Metal Delivery System

The apparent solubility study was assayed in a paddle apparatus (Agilent Technologies, Santa Clara, CAL USA). Gelatin capsules were carefully filled with CA (10.0 mg), 10.0 mg of CA and Neu physical mixture (CA_Neu_1:1_PHM), and their 20 min ball milled system (CA_Neu_1:1_BM_20′), and placed in springs to prevent floating.

The capsules were positioned in vessels containing 500 mL of two different media: hydrochloric acid with a pH of 1.2, and phosphate buffer with a pH of 6.8. The temperature was adjusted to 310.15 K and the paddles rotated at a speed of 50 rpm. The study was carried out for 240 min; the withdrawal sampling times were 5 min, 10 min, 15 min, 30 min, 45 min, 60 min, 90 min, 120 min, 180 min, and 240 min. During sampling points, 2.0 mL samples were withdrawn and instantly substituted with an equivalent volume of the temperature-equilibrated fresh medium. Later, filtration of the samples was performed using a 0.22 μm membrane filter and analyzed by HPLC. The variations and resemblances in the profiles were evaluated using the two-factor values *f*_1_ and *f*_2_ developed by Moore and Flanner [77]. These values were calculated using the following equations:

(1) $$f_{1}=\frac{\sum_{j=1}^{n}\left|R_{j}-T_{j}\right|}{\sum_{j=1}^{n}R_{j}}$$

(2) $$f_{2}=50\times \log\left({\left(1+\left(\frac{1}{n}\right)\overset{n}{\underset{j=1}{\sum }}{\left|R_{j}-T_{j}\right|}^{2}\right)}^{-\frac{1}{2}}\times 100\right)$$

In the equations, *n* represents the total number of time points considered. *R_j_* denotes the percentage of the reference dissolved substance in the medium at a specific time point, while *T_j_* represents the percentage of the dissolved tested substance at the same time point. The variable t signifies the specific time point. Dissolution profiles are determined as similar when the *f*_1_ value is close to 0 or *f*_2_ is close to 100 (between 50 and 100) [78].

### 3.8. Gastrointestinal and Blood-Brain Barrier Membranes Permeability of the CA from the Inorganic Metal Delivery System

The CA permeability through biological membranes was studied with the use of the PAMPA models in the GIT (pH 1.2 and 6.8) and BBB (pH 7.4) models. The study was performed in two 96-well microfilter plates, with donor chambers at the bottom and acceptor at the top separated by a 120 μm-thick microfilter disc coated with a 20% (*w*/*v*) dodecane solution of a lecithin mixture (Pion Inc., Billerica, MA, USA). The CA and CA physical mixture and solid dispersion were dissolved in DMSO and placed in donor solutions which were adjusted to pH ≈ 1.2 and 6.8 for the GIT model and to pH ≈ 7.4 for BBB. Taking into consideration the fact that magnesium aluminometasilicate does not enter the bloodstream, the BBB permeability was only studied for CA, as Neu does not impact its permeability outside GIT. The plates were incubated in a humidity-saturated atmosphere, with the temperature set at 310.15 K for 3 h for the GIT assay and 4 h for the BBB model. Subsequently, the plates were separated and CA concentrations were determined using the HPLC-DAD method. The *P_app_* value was calculated using the following equations:

(3) $$P_{app}=\frac{-ln\left(1-\frac{C_{A}}{C_{equilibrium}}\right)}{S\times \left(\frac{1}{V_{D}}+\frac{1}{V_{A}}\right)\times t}$$

(4) $$C_{equilibrium}=\frac{C_{D}\times V_{D}+C_{A}\times V_{A}}{V_{D}+V_{A}}$$

where: *V_D_*—donor volume, *V_A_*—acceptor volume, *C_equilibrium_*—equilibrium concentration $C_{equilibrium}=\frac{C_{D}\times V_{D}+C_{A}\times V_{A}}{V_{D}+V_{A}}$, *S*—membrane area, *t*—incubation time (in seconds).

Compounds with a *P_app_* in the GIT model below 0.1 × 10^−6^ cm s^−1^ are determined as poorly permeable, APIs with 0.1 × 10^−6^ cm s^−1^ ≤ *P_app_* < 1 × 10^−6^ cm/s are classified as medium permeable, and compounds which are highly permeable have a *P_app_* ≥ 1 × 10^−6^ cm s^−1^ [79]. Substances with *P_app_* in the BBB model < 2.0 × 10^−6^ cm s^−1^ are described as poorly permeable. APIs with *P_app_* values in the range of 2.0 to 4.0 × 10^−6^ cm s^−1^ are determined to have questionable permeability. Substances with good permeability have a *P_app_* value at the level of >4.0 × 10^−6^ cm s^−1^ [80].

### 3.9. Biological Activity of the CA from the Inorganic Metal Delivery System

This study aimed to determine the impact of solid dispersion system formation on CA neuroprotective activity. Neu is not soluble in water, does not leave GIT to enter the bloodstream, and does not have any impact outside GIT; thus, in order to avoid introducing a false impact into the final results, the following steps were chosen in all biological activity models. The graphs of the dependence of biological activity on the concentration of CA were prepared. Later, the exact results for the concentrations equal to the solubility of CA and CA in the systems were determined. Subsequently, the biological activity of pure CA and CA activity resulting from the preparation of the systems was determined.

#### 3.9.1. Antioxidant Activity

CA antioxidant activity was determined by studying its ability to scavenge radicals in DPPH, ABTS techniques, and the ability to reduce ions in the FRAP model.

The reaction between (0.2 mM) methanol solution of DPPH and CA and its systems was evaluated spectrophotometrically [73]; 25.0 µL of increasing CA concentrations from 50.0 μg/mL to 1000.0 μg/mL were mixed with 175.0 µL of the DPPH solution on a 96-well plate and incubated while shaking for 30 min in dark conditions at room temperature, then measured on a plate reader (Multiskan GO, Thermo Fisher Scientific, Waltham, MA, USA) at 517 nm. The absorbance (A) was measured for the blank (mixture of DPPH solution and DPPH) at 517 nm. Inhibition of DPPH radicals was calculated using the following formula:

(5) $$A=\frac{A_{0\;}-A_{1}}{A_{0}}\times 100\%$$

where *A*_0_ is the absorbance of the control sample and *A*_1_ is the absorbance of the studied sample. Each measurement was repeated six times. Trolox (6-hydroxy-2,5,7,8-tetramethylchroman-2-carboxylic acid, a vitamin E analogue) was used as a reference. IC_50_ values for determining the concentration of a compound or extract that inhibits DPPH formation by 50%, were determined by linear regression analysis.

For ABTS, the second method evaluating scavenging radical properties was performed according to Re et al. [81] and modified. Green cation radicals are produced by the loss of electrons by the nitrogen atoms of ABTS caused by potassium persulfate. After introducing the preformed radical cation into the antioxidant, the ABTS radical cation is reduced back to its colorless neutral form. The concentrations of CA for the assay were prepared in 50% DMSO ranging from 26.67 μg/mL to 173.33 μg/mL; 10.0 μL of CA dilutions and 200.0 μL of ABTS^•+^ solution were added to 96-well plates. Subsequently, the plates were incubated while shaking for 10 min at room temperature. After incubation, absorbance values were measured at λ = 734 nm (Multiskan GO, Thermo Fisher Scientific, Waltham, MA, USA). Trolox was used as a standard. The inhibition of ABTS^•+^ by CA was calculated using the following equation:

(6) $$\mathrm{ABTS}\;\mathrm{scavenging}\;\mathrm{activity}\;\left(\%\right)=\frac{A_{0}-A_{1}}{A_{0}}\times 100\%$$

where:

*A*_0_—the absorbance of the control

*A*_1_—the absorbance of the sample

The ability to reduce ions was tested in a Ferric Ion Reducing Antioxidant Parameter (FRAP) Assay, which is based on the reduction of colorless Fe^3+^ ions to Fe^2+^ with the simultaneous formation of a dark blue complex with TPTZ (2,4,6-tris(2-pyridyl)-1,3,5-triazine) [82]. CA (25.0 µL) was added pipetted to the 96-well plate with FRAP mixture (25 mL acetate buffer, 2.5 mL FeCl_3_·6H_2_O solution, and 2.5 mL TPTZ solution) and incubated for 30 min at 310.5 K in the dark. The absorbance was measured at the wavelength λ = 593 nm. (Multiskan GO, Thermo Fisher Scientific, Waltham, MA, USA). Six replicates were used in the analysis, and Trolox was used as a standard reference. The IC_0.5_ value is the CA concentration indicating 0.5 absorbance.

#### 3.9.2. Determination of Enzymes Influencing the Development of Neurodegenerative Diseases Inhibition

The AChE and BChE inhibition was studied using a spectrometric Ellman et al. modified assay [83]. This method requires artificial substrates (thiocholine esters). Thiocholine is liberated during the enzymatic reactions with 5,5′-dithio-bis-(2-nitrobenzoic) acid (DTNB), and the 3-carboxy-4-nitrothiolate anion (TNB anion) is formed.

The CA concentrations for the assay were prepared in the range of 0.3 mg/mL to 20.0 mg/mL in DMSO in the AChE inhibition assay and from 0.3 to 17.33 mg/mL in the BChE inhibition method. The enzyme activity technique is based on spectrophotometrical measurement according to the elevation in the thiocholine color in a 96-well plate. The wells contained 0.05 M Tris-HCl buffer (60.0 μL) with a pH of 8.0, test solution (20.0 μL), and AChE/BChE solution (30.0 μL) at a concentration of 0.2 U/mL. The plates were incubated while shaking for 5 min at room temperature. Subsequently, 1.5 mM acetylthiocholine iodide (ATCI)/butyrylthiocholine iodide (BTCI) solution (30.0 μL) and 0.3 mM DTNB solution (5,5′-dithiobis-(2-nitrobenzoic acid) (125.0 μL) were pipetted to the plate and incubated under equal conditions for 20 min. A blank for the test sample (the reaction mixture was stripped depleted of the enzyme, and the volume of Tris-HCl buffer was raised), the control sample (solvent was added instead of the test sample), and a blank for the control sample (the reaction mixture of the control sample was stripped of the enzyme (the volume of Tris-HCl buffer was raised)) were prepared as well. Galantamine was used as a reference,. The absorbance was measured at the wavelength of 405 nm. The percentage of inhibition of AChE and BChE by the samples was calculated according to the following equation:

(7) $$\mathrm{AChE}/\mathrm{BChE}\;\mathrm{inhibition}\;\left(\%\right)=\frac{1-\left(A_{1}-A_{1b}\right)}{\left(A_{0}-A_{0b}\right)}\times 100\%$$

where:

*A*_1_—the absorbance of the test sample

*A*_1*b*_—the absorbance of the blank of the test sample

*A*_0_—the absorbance of control

*A*_0*b*_—the absorbance of the blank of control

The tyrosinase inhibition assay involves the use of L-DOPA, an amino acid that helps restore dopamine levels, as a substrate for the tyrosinase enzyme. Tyrosinase facilitates the conversion of tyrosine to L-DOPA and L-DOPA to dopaquinone. When tyrosinase is inhibited, the breakdown of L-DOPA, which serves as a precursor to dopamine, is reduced. This inhibition is beneficial for individuals with Parkinson’s disease, as they have insufficient levels of dopamine. The decrease in solution color intensity is due to the inhibition of enzyme activity, which is the basis of the assay [84]. The inhibitor blocks L-DOPA access to the tyrosinase active site, which prevents the reaction from proceeding. In this assay, CA was dissolved in DMSO in ascending concentrations from 0.25 mg/mL to 10.0 mg/mL. The assay was performed on 96-well plates. The test sample contained 0.1 M phosphate buffer at a pH of 6.8 (75.0 μL), test solutions (25.0 μL), and enzyme solution (192 U/mL) (50.0 μL). The samples were incubated at room temperature for 10 min while shaking. After incubation, 2.0 mM L-DOPA (50 μL) was pipetted to the wells and incubated for another 20 min under the same conditions. A blank for the test sample (the reaction mixture was depleted of the enzyme, and the volume of phosphate buffer was enlarged), the control sample (solvent was used instead of the test sample), and a blank for the control sample (the reaction mixture of the control sample was stripped of the enzyme (the volume of phosphate buffer was raised) were prepared as well.

The absorbance of the test samples was assessed at a specific wavelength of 475 nm, with azelaic acid serving as a reference standard. The percentage of tyrosinase inhibition by the samples was determined using the following formula:

(8) $$\mathrm{Tyrosinase}\;\mathrm{inhibition}\;\left(\%\right)=\frac{1-\left(A_{1}-A_{1b}\right)}{\left(A_{0}-A_{0b}\right)}\times 100\%$$

where:

*A*_1_—the absorbance of the test sample

*A*_1*b*_—the absorbance of the blank of test sample

*A*_0_—the absorbance of control

*A*_0*b*_—the absorbance of the blank of control

#### 3.9.3. Molecular Docking Study

The docking program MGLTools 1.5.6 with AutoDock 4.2 (ADT; Scripps Research Institute, La Jolla, San Diego, CA, USA) [85] was used to explore the probable interaction between CA and tyrosinase. The three-dimensional (3D) structures of tyrosinase (PDB ID: 2Y9X) were downloaded from RCSB Protein Data Bank (https://www.rcsb.org/, accessed on 15 December 2022). The structure of CA (PubChem CID: 689043) was retrieved from PubChem (https://pubchem.ncbi.nlm.nih.gov/, accessed on 15 December 2022).

The ligands and water molecules were removed from the original Protein Data Bank file. AutoDock Tools was used to add polar hydrogen atoms and Kollman charges. In addition, missing atoms were corrected and atomic Gasteiger charges were calculated and added. The scoring grid box for tyrosinase was reported previously [86]; its parameters are X-center: −8.064, Y-center: −25.776, Z-center: −39.384; grid size: 60 × 60 × 60 points, and the spacing value: 0.375 Å. The grid maps for energy scoring were calculated using AutoGrid. Docking calculations were performed using the Lamarckian genetic algorithm, and the search parameters were set to 100 times. After docking simulations, the best scores (i.e., with the lowest docking energy) were selected and exported to the PDBQT format. Open Babel program (http://openbabel.org, accessed on 17 December 2022) was used to convert the PDBQT file to the PDB format [87]. The Protein-Ligand Interaction Profiler (https://plip-tool.biotec.tu-dresden.de/, accessed on 17 December 2022) was used to analyze the resulting interactions and export the file to the PyMol format [88]. PyMol 2.5.1 (DeLano Scientific LLC, Palo Alto, CA, USA) was used to save the visualization [89].

### 3.10. Statistical Analysis

Statistical analysis was performed using Statistica 13.3 software from StatSoft (StatSoft Poland, Krakow, Poland) The data were expressed as mean values with standard deviations. Normality of the distributions was assessed using skewness and kurtosis tests, while equality of variances was evaluated using Levene’s test. Statistical significance was determined using a one-way analysis of variance (ANOVA) followed by the Bonferroni post hoc test or by or the Kruskal–Wallis test (to compare the experimental results acquired for CA and CA in the systems). Differences were considered significant at *p* < 0.05.

## 4. Conclusions

The applied mechanosynthesis techniques based on the use of magnesium aluminometasilicate allowed for a significant improvement in the solubility of caffeic acid. This is the result of salt formation during ball milling and lyophilization between the caffeic acid and ions present in the used carrier. Additionally, the tendency to decrease the contact area of caffeic acid with silicate was important.

The benefits of improving the solubility of caffeic acid include better permeability through biological membrane systems simulating the walls of the digestive system and the blood–brain barrier. Therefore, the significant neuroprotective effect expressed in the inhibition of enzymes that impact the development of neurodegenerative diseases such as acetylcholinesterase, butyrylcholinesterase, and tyrosinase and the possibility of scavenging free radicals for caffeic acid from metal in-organic delivery systems can be extrapolated to in vivo conditions.

## Figures and Tables

**Table 1:** Gastrointestinal (GIT) and blood-brain (BBB) permeability coefficient values of caffeic acid (CA), CA and Neusilin US2 (Neu) physical mixture (CA_Neu_1:1_PHM), and solid dispersion obtained by 20 min of ball milling (CA_Neu_1:1_BM_20′) in a pH of 1.2 and 6.8. (*) indicates statistically significant differences, *p* < 0.05.

The Substance/System	Permeability Coefficient (cm s^−1^)		
	pH 1.2	pH 6.8	BBB
CA	7.405 × 10^−6^ ± 2.872 × 10^−7^	1.445 × 10^−7^ ± 3.486 × 10^−8^	1.142 × 10^−6^ ± 2.006 × 10^−7^
CA_Neu_1:1_PHM	7.546 × 10^−6^ ± 7.741 × 10^−8^	1.322 × 10^−7^ ± 2.431 × 10^−8^	-
CA_Neu_1:1_BM_20′	8.850 × 10^−6^ ± 1.765 × 10^−7^ (*)	1.812 × 10^−7^ ± 8.183 × 10^−9^	-

**Table 2:** Inhibition of acetylcholinesterase (AChE), butyrylcholinesterase (BChE), and tyrosinase by caffeic acid (CA), CA and Neusilin US2 (Neu) physical mixture (CA_Neu_1:1_PHM), and solid dispersion obtained by 20 min of ball milling (CA_Neu_1:1_BM_20′). N/D—not detectable. (*) indicates statistically significant differences, *p* < 0.05.

The Substance/System	Enzyme Inhibitory Potential		
	AChE	BChE	Tyrosinase
CA	N/D	N/D	2.657 ± 0.039%
CA_Neu_1:1_PHM	11.854 ± 0.396% (*)	8.644 ± 0.121% (*)	29.443 ± 0.150% (*)
CA_Neu_1:1_BM_20′	18.444 ± 0.429% (*)	13.011 ± 0.209% (*)	58.658 ± 0.173% (*)

## Data Availability

Data are available in a publicly accessible repository.

## Supplementary Materials

The following supporting information can be downloaded at: https://www.mdpi.com/article/10.3390/ijms24119218/s1.

## Abbreviations

ABTS	2,2′-azino-bis(3-ethylbenzothiazoline-6-sulfonic acid)
AChE	Acetylcholinesterase
ANOVA	one-way analysis of variance
ATCI	acetylthiocholine iodide
ATR-FTIR	Attenuated Total Reflectance Fourier Transform Infrared spectroscopy
BBB	blood-brain barrier
BChE	Butyrylcholinesterase
BM	ball milling
BTCI	butyrylthiocholine iodide
CA	caffeic acid
CD	Cyclodextrin
CUPRAC	cupric reducing antioxidant capacity
DMSO	dimethyl sulfoxide
DPPH	2,2-diphenyl-1-picrylhydrazyl
DTNB	5,5′-dithio-bis-(2-nitrobenzoic) acid
EUD.L100	eudragit L100
FD	freeze-drying
FRAP	ferric reducing antioxidant power
GIT	gastrointestinal tract
GOH	gohsenol EG-05PW
HPLC	high-performance liquid chromatography
HP-β-CD	2-hydroxypropyl-β-cyclodextrin
KOLL.VA64	kollidon VA 64
NEU	neusilin US2
PAMPA	parallel artificial membrane permeability assay
PHM	physical mixture
RPM	rotations per minute
TNB	3-carboxy-4-nitrothiolate
TPTZ	2,4,6-tris(2-pyridyl)-1,3,5-triazine
XRPD	X-ray Powder Diffraction
