# Supplementary material for: Screening of the Anti-Neurodegenerative Activity of Caffeic Acid after Introduction into Inorganic Metal Delivery Systems to Increase Its Solubility as the Result of a Mechanosynthetic Approach
Source: Int J Mol Sci. 2023 May 24;24(11):9218. doi: 10.3390/ijms24119218 (PMC10252749; doi:10.3390/ijms24119218)
Supplement: Supplementary file 1 [file ijms-24-09218-s001.zip › ijms-2351526-supplementary.pdf]

Table S1. Selected solubility values (µg/mL) of CA, freeze-dried and ball milled (after 20 min) CA; solubility values of the physical mixtures 1:1 and 1:3 mass ratio with excipients; selected solubility values of CA and freeze-dried and ball milled (after 20 min) solid dispersions in mass ratio 1:1 and 1:3. * indicated statistic significance; Abbreviations: HP-β-CD - hydroxypropyl-β-cyclodextrin, Koll.VA64 - Kollidon VA 64, Eud.L100 Eudragit L100, Goh - Gohsenol EG-05PW, Neu - Neusilin US2, phm – physical mixture, FD – freeze dried, BM – ball milled.

Substance/solid dispersion	Solubility (µg/mL)	
	Mass ratio	
	1:1	1:3
CA	303.274 ± 6.218	
CA_HP-β-CD_PHM	1232.670 ± 3.341*	2022.361 ± 20.223*
CA_Koll.VA64_PHM	1067.618 ± 6.911*	1993.973 ± 19,939*
CA_Neu_PHM	2535.204 ± 5.352*	3281.838 ± 15.283*
CA_ Goh _PHM	330.888 ± 3.520	359.415 ± 7.543
CA_Eud.L100_PHM	345.968 ± 1.602	365.584 ± 6.345
CA_FD	302.164 ± 5.579	
CA_HP-β-CD_FD	1298.020 ± 1.794*	2262.34 ± 14.766*
CA_Koll.VA64_FD	916.859 ± 6.338*	758.203 ± 2.071*
CA_Neu_FD	2605.226 ± 18.166	3007.422 ± 17.653*

CA_Goh_FD	382.914 ± 7.105	404.325 ± 4.058*
CA_Eud.L100_FD	388.755 ± 6.145	401.663 ± 6.532*
CA_BM_20'	309.086 ± 5.476	
CA_HP-β-CD_BM_20'	1139.644 ± 4.755*	2077.756 ± 20.777*
CA_Koll.VA64_BM_20'	953.609 ± 3.294*	1580.101 ± 15.800*
CA_Neu_BM_20'	**5449.017 ± 19.420***	3725.359 ± 8.755*
CA_Goh_BM_20'	312.335 ± 3.304	331.415 ± 4.205
CA_Eud.L100_BM_20'	309.454 ± 2.316	329.544 ± 3.646

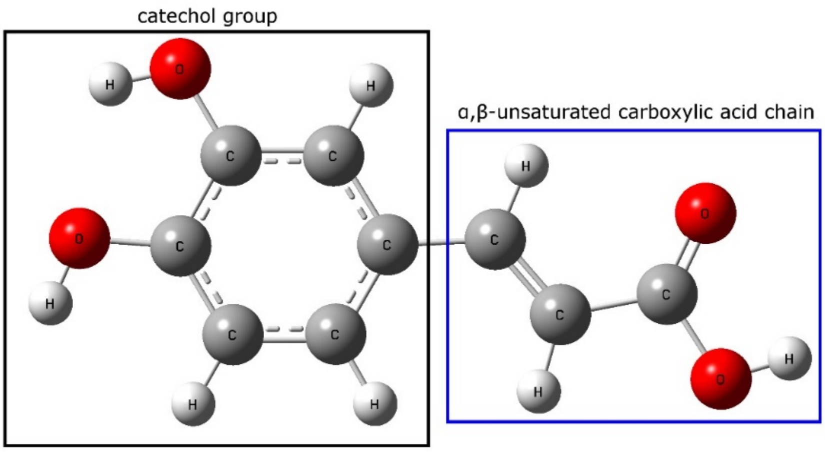

Figure S1. The optimized geometry of the caffeic acid, containing a catechol group with an α,β-unsaturated carboxylic acid chain.

Table S2. Selected experimental and theoretical modes (in cm^{-1}) characteristic vibronic features of caffeic acid. Legend: r–rocking, s–stretching, w–wagging, def.–deformation, side chain - α,β-unsaturated carboxylic acid chain

DFT [cm^{-1}]	EXP [cm^{-1}]	Band assignment
424	457	O–H w in catechol group
512	550<br>575	def. all molecule
568	588	COOH b + def. benzene ring
580	602	C–H w at side chain and benzene ring + O–H w in side chain
632	646<br>698	O–H w in side chain + CCC t in benzene ring + C–H w at benzene ring
656	737<br>779	COOH b + CCC b in all molecule
752	800<br>814	C–H w in all molecule + C–O–H w in side chain + CCC t in side chain
800	849	C–OH s in catechol group + CCC s in benzene ring or C–H w at benzene ring
872	893	C–H w at benzene ring and side chain
976	935	C–H r in all molecule + O–H b in side chain
1032	968	C–H w at side chain
1152	1119	C–H r in all molecule + O–H b in side chain
1224	1173	C–O–H b in catechol group + C–H r at benzene ring + CC s in benzene ring
1272	1213	C–H b at side chain + O–H b in side chain
1320	1273	breathing benzene ring + C–H r in all molecule + C–O–H b in side chain + O–H b in catechol group
1352	1294	breathing benzene ring + C–H r at side chain + O–H b in side chain
1408	1352	CC s in benzene ring + O–H b in all molecule
1504	1375	CC s in benzene ring + O–H b catechol group
1568	1447	CC s in benzene ring
1648	1522	CC s in benzene ring and C=C s in side chain + O–H b in catechol group
1664	1599	CC s in benzene ring and C=C s in side chain + O–H b in catechol group
1696	1616	CC s in benzene ring and C=C s in side chain + O–H b in catechol group
1808	1641	C=O s and O–H b in side chain
3768	3217	O–H s in side chain
3784	3402	O–H s in catechol group
3840	3428	O–H s in catechol group
